# Imaging findings of diabetic complications from head to toe: a comprehensive radiologic review

**DOI:** 10.1007/s11604-026-01988-6

**Published:** 2026-04-16

**Authors:** Fumiko Yagi, Yoshitake Yamada, Kazuma Yagi, Hirotaka Akita, Masahiro Jinzaki

**Affiliations:** 1 Department of Diagnostic Radiology, Keio University School of Medicine, 35 Shinanomachi, Shinjuku, Tokyo 160-8582 Japan; 2 Division of Diabetes, Metabolism and Endocrinology, Department of Internal Medicine, Sainokuni Higashiomiya Medical Center, Saitama, Japan

**Keywords:** Diabetes mellitus, Computed tomography, Magnetic resonance imaging, Infectious complications, Metabolic disorders

## Abstract

Diabetes mellitus is a systemic metabolic disease that causes a wide spectrum of complications affecting multiple organs. Advances in imaging modalities have substantially improved the detection, characterization, and clinical management of these conditions. However, the imaging manifestations of diabetic complications are highly diverse, often mimicking other diseases and posing diagnostic challenges for radiologists. This review aimed to provide an organ-based overview of the characteristic imaging features of diabetic complications from head to toe, integrating pathophysiological mechanisms relevant to image interpretation. Representative cases are illustrated using multimodal imaging—computed tomography (CT), magnetic resonance imaging, ultrasound, radiography, and ^18^F-fluorodeoxyglucose positron emission tomography/CT—to highlight key findings, differential diagnoses, and potential pitfalls. Specifically, this review focuses on conditions and diseases that are amenable to evaluation by radiological imaging. Understanding these imaging patterns is essential for early diagnosis, risk stratification, and optimal patient management. Through an educational, pictorial approach, this review aims to enhance radiologists’ recognition of diabetic complications across organ systems.

## Introduction

Diabetes mellitus (DM) is a global health problem characterized by chronic hyperglycemia and multisystem involvement. According to the International Diabetes Federation, its prevalence continues to increase worldwide, increasing the burden of diabetes-related complications [1].

Pathophysiologically, persistent hyperglycemia, insulin resistance, and chronic low-grade inflammation contribute to metabolic derangements, leading to characteristic structural and functional changes that can be detected via imaging studies, often before overt clinical symptoms develop. Recognizing these findings enables early risk stratification and clinical intervention. Furthermore, imaging is essential in the detection, characterization, and differentiation of diabetic complications. Because many imaging findings are nonspecific and may mimic malignancy or severe infection, radiologists must be familiar with typical imaging patterns and common diagnostic pitfalls. This review aimed to provide an organ-based overview of diabetic complications, focusing on characteristic imaging findings and clinically relevant differential diagnoses.

## Pathophysiology relevant to imaging

Diabetic complications arise from a complex interplay of metabolic, vascular, and immunologic disturbances that progressively affect multiple organ systems [2, 3]. Understanding these underlying mechanisms is essential for radiologists, as many imaging findings directly reflect characteristic pathophysiological processes.

Chronic hyperglycemia is the central driver of diabetic tissue injury. Persistent elevation of glucose levels leads to the formation of advanced glycation end products, oxidative stress, and endothelial dysfunction, which accelerate capillary basement membrane thickening and microangiopathy, particularly in the kidneys, retina, and peripheral nerves. Macrovascular disease is accelerated by dyslipidemia, inflammation, and vascular smooth muscle dysfunction, predisposing patients to coronary artery stenosis, limb ischemia, and cerebrovascular infarction.

Insulin resistance significantly contributes to structural changes observed on imaging. Impaired insulin signaling causes fat deposition in metabolically vulnerable organs—including the liver and pancreas [4, 5]. These alterations manifest radiologically as hepatic steatosis and pancreatic fatty infiltration, each associated with functional impairment. Fat accumulation also enhances inflammatory pathways that exacerbate cardiovascular risk and metabolic instability [5].

Immune dysfunction represents another key pathophysiologic component. Neutrophil chemotaxis, macrophage activation, and cellular immunity are all impaired in diabetes, reducing host defense mechanisms [2]. Combined with microvascular insufficiency, this immune deficit predisposes patients to atypical, fulminant infections such as emphysematous cholecystitis, emphysematous pyelonephritis (EPN) [6], necrotizing fasciitis [7], and Fournier gangrene [8]. These conditions demonstrate distinctive imaging features—including gas formation within tissues—essential for urgent diagnosis and management.

Neurologic complications also stem from metabolic and vascular disturbances. Rapid fluctuations in serum glucose may cause transient metabolic dysfunction, exemplified by non-ketotic hyperglycemic hemichorea with T1 hyperintensity in the basal ganglia [9]. Conversely, prolonged hypoglycemia results in diffuse cytotoxic edema, particularly in the cerebral cortex, hippocampus, and basal ganglia [10].

Moreover, certain anti-diabetic medications can produce specific imaging patterns. Metformin is associated with diffuse bowel uptake on ¹⁸F-fluorodeoxyglucose positron emission tomography/computed tomography (FDG-PET/CT) [11], while sodium–glucose cotransporter 2 (SGLT2) inhibitors may influence susceptibility to urogenital infections. Understanding these drug-related appearances helps avoid misinterpretation and unnecessary interventions.

An understanding of these interconnected mechanisms enables radiologists to more accurately interpret a wide spectrum of imaging findings in patients with diabetes and to distinguish true pathological abnormalities from mimics or incidental variations.

### Imaging modalities for evaluating diabetic complications

Multiple imaging modalities play complementary roles in evaluating diabetic complications. Computed tomography (CT) is particularly valuable in acute settings, enabling rapid detection of emphysematous infections [12], calcifications, and soft-tissue gas in necrotizing infections [12].

Magnetic resonance imaging (MRI) provides superior soft-tissue contrast and functional assessment, allowing detailed evaluation of neurological injuries [9, 10], myocardial involvement [13], and musculoskeletal infections [7]. Advanced techniques, including diffusion-weighted imaging and chemical shift imaging, facilitate the detection of cytotoxic edema and fatty infiltration.

Ultrasonography remains a practical first-line modality for assessing hepatobiliary, renal, and reproductive organ abnormalities, whereas FDG-PET/CT offers metabolic information on inflammatory or infectious conditions. Awareness of characteristic drug-related imaging findings, particularly metformin-associated bowel uptake, is essential [11]. Notably, integrating multimodal imaging within the clinical context enables accurate diagnosis and appropriate management of diabetic complications.

## Organ-based review of diabetic complications

### Head and neck

#### Malignant otitis externa (Fig. 1)

Malignant otitis externa is a severe, invasive infection of the external auditory canal that predominantly affects older patients with long-standing DM [14], caused by impaired host immunity and microvascular insufficiency, allowing the infection to extend to the temporal bone and skull base, causing skull base osteomyelitis. The causative pathogen is usually *Pseudomonas aeruginosa*. Early diagnosis is critical because delayed diagnosis may lead to cranial neuropathies and life-threatening intracranial complications. CT is useful for detecting cortical bone erosion of the external auditory canal and adjacent skull base structures, whereas MRI is essential in evaluating bone marrow involvement, soft-tissue extension, and intracranial spread [15]. On MRI, inflammatory changes typically appear as low signal intensity on T1-weighted images and high signal intensity on fluid-sensitive sequences, with contrast enhancement, respectively [15].

A major diagnostic pitfall is confusion with malignant neoplasms of the skull base, such as nasopharyngeal carcinoma or metastatic disease, as both entities demonstrate aggressive bone destruction and infiltrative soft-tissue masses. However, malignant otitis externa usually shows diffuse inflammation without a discrete mass. Correlation with clinical findings—refractory otitis externa, otalgia, and elevated inflammatory markers—is essential for accurate diagnosis. Persistent imaging abnormalities despite clinical improvement should not be misinterpreted as treatment failure, as radiologic resolution often lags behind clinical response.

### Intracranial infections in diabetes

Patients with diabetes mellitus, particularly those with poor glycemic control or ketoacidosis, are at greater risk for invasive intracranial infections, most notably rhino-orbito-cerebral mucormycosis [16]. This angioinvasive fungal infection typically originates in the sinonasal cavity and may extend to the orbit and intracranial compartment. CT/MRI may demonstrate sinonasal soft-tissue infiltration with extrasinus extension, and intracranial complications such as cavernous sinus involvement, internal carotid artery invasion/thrombosis, cerebral infarction, and abscess formation. Early recognition of these imaging findings is crucial because prompt antifungal therapy and surgical debridement are often required.

### Central nervous system

#### Non-ketotic hyperglycemic hemichorea (Fig. 2)

Non-ketotic hyperglycemic hemichorea is a rare neurologic complication of poorly controlled DM, typically affecting older patients [9, 17]. It results from hyperglycemia-related metabolic derangement of the basal ganglia without ketoacidosis, causing unilateral choreiform or ballistic movements [9]. Prompt recognition is important, as symptoms often resolve rapidly with glycemic control.

On imaging, CT may show subtle hyperattenuation of the contralateral basal ganglia, while MRI classically demonstrates unilateral hyperintensity on T1-weighted images involving the putamen and caudate nucleus [9]. T2-weighted and diffusion-weighted images show variable signal changes, usually without mass effect [9]. Its pathophysiology remains unclear but likely involves metabolic dysfunction, petechial hemorrhage, and protein desiccation within the basal ganglia.

A common diagnostic pitfall is misinterpreting T1 hyperintensity as hemorrhage or calcification. However, the lack of susceptibility artifacts on gradient-echo or susceptibility-weighted imaging, absence of surrounding edema, and rapid clinical improvement with glycemic control suggest a metabolic etiology. Awareness of this condition helps prevent unnecessary invasive investigations and inappropriate management.

#### Hypoglycemic encephalopathy (Fig. 3)

Hypoglycemic encephalopathy is a potentially devastating DM complication caused by prolonged or severe hypoglycemia, often associated with insulin or oral hypoglycemic agents [17]. Neurons in the cerebral cortex and deep gray matter are particularly vulnerable, leading to cytotoxic edema and characteristic imaging findings. MRI is the most sensitive modality, typically demonstrating bilateral symmetric hyperintensity on diffusion-weighted imaging with low apparent diffusion coefficient values [10]. The cerebral cortex, hippocampus, basal ganglia, and sometimes the cerebellum may be involved, whereas the brainstem is relatively unaffected [10]. Fluid-attenuated inversion recovery (FLAIR) images may exhibit delayed signal changes following diffusion abnormalities. Similar changes occur with hypoxia, hyperammonemia, encephalitis, seizures, or may be drug-induced [17]. A key diagnostic challenge is differentiation from hypoxic–ischemic encephalopathy [17, 18], which more often shows diffuse cortical and deep gray matter involvement, including the thalami, basal ganglia, and brainstem—particularly in severe cases [18]. Clinical history, including documented hypoglycemia and temporal correlation with symptoms, is therefore crucial. Early recognition is essential, as diffusion patterns may have prognostic implications.

#### Stroke (Fig. 4)

DM is a well-established risk factor for both macrovascular and microvascular cerebrovascular diseases [3]. Chronic hyperglycemia accelerates atherosclerosis and promotes endothelial dysfunction, increasing the risk of ischemic stroke, extensive cerebral white matter changes, recurrent strokes, and poorer functional outcomes [19]. Diabetes is also associated with silent infarctions and cerebral small-vessel disease, including white matter hyperintensities and lacunes [20]. MRI is central to stroke evaluation. Acute ischemic infarction is best detected using diffusion-weighted imaging [21], whereas FLAIR images reveal chronic white matter hyperintensities, reflecting microvascular disease [22].

### Diabetes-related cognitive impairment and dementia

DM is associated with cognitive impairment and dementia due to chronic microvascular injury and metabolic dysfunction. Imaging findings are nonspecific, often overlapping with other neurodegenerative conditions, and are therefore not diagnostic.

### Cardiovascular system

#### Diabetic cardiomyopathy (Fig. 5)

Diabetic cardiomyopathy is characterized by structural and functional myocardial abnormalities occurring independently of coronary artery disease (CAD) or hypertension. Chronic hyperglycemia, insulin resistance, microvascular dysfunction, and myocardial fibrosis exacerbate impaired diastolic and systolic functions [23]. This condition may progress silently and predispose patients to heart failure even in the absence of overt ischemic heart disease. Cardiac MRI is central to evaluation [13], demonstrating increased native T1 values, expanded extracellular volume, and patchy mid-wall late gadolinium enhancement, reflecting diffuse interstitial fibrosis [13, 24]. These changes do not follow a coronary distribution and may precede functional impairment on echocardiography. A key diagnostic pitfall is misattributing myocardial fibrosis to ischemic heart disease. CT is used to screen for and rule out CAD [13]. Careful assessment of the coronary anatomy and clinical history is essential to avoid misdiagnoses.

#### Coronary artery disease (CAD) (Fig. 6)

DM is a well-established risk factor for CAD. Prospective cohort studies, including the Framingham Heart Study, have demonstrated a 2–3-fold increased risk of incident CAD in individuals with diabetes, even after adjusting for traditional cardiovascular risk factors [25]. CAD in patients with DM is characterized by diffuse, multivessel involvement and a higher total plaque burden than in non-diabetic patients [26]. On coronary CT angiography, patients with diabetes more frequently demonstrate non-calcified or mixed plaques and high-risk plaque features, even with mild-to-moderate luminal stenosis [27]. Coronary artery calcification scores are often elevated; however, vulnerable non-calcified plaque components commonly coexist [27]. Silent ischemia and multifocal perfusion abnormalities are also more prevalent, underscoring the importance of comprehensive imaging assessment beyond symptom-based evaluation [27].

#### Peripheral artery disease (PAD) (Fig. 7)

PAD is a major macrovascular complication of diabetes mellitus and is associated with chronic limb-threatening ischemia (CLTI), amputation, and cardiovascular mortality [28, 29]. Since diabetic neuropathy may mask claudication, patients may present late with rest pain, non-healing ulcers, or tissue loss. Imaging is essential for confirming disease extent and planning revascularization: duplex ultrasonography is often used initially, whereas CT angiography (CTA), MR angiography (MRA), and digital subtraction angiography (DSA) delineate stenosis/occlusion severity, lesion length, runoff vessels, and collateral pathways [28–30]. In diabetes, heavy (often infrapopliteal) calcification can limit luminal assessment on CTA; careful interpretation with modality correlation may be required [30]. Medial arterial calcification (Mönckeberg’s sclerosis), frequently seen in diabetes and chronic kidney disease, may also cause falsely elevated ankle–brachial index values and should be recognized when integrating physiologic tests with imaging. In addition, radiologists should evaluate limb-threatening comorbidities (ulceration, osteomyelitis, soft-tissue infection) that influence urgency and strategy of intervention, and note the patency of the fibular (peroneal) artery as a potential collateral pathway [29].

### Lung

#### Coronavirus disease 2019 (COVID-19) (Fig. 8)

Severe COVID-19 in patients with DM is clinically important because diabetes is associated with worse clinical outcomes and has been encountered in daily imaging practice [31]. Chest CT in COVID-19 typically demonstrates bilateral, peripheral-predominant ground-glass opacities with or without consolidation, sometimes with a crazy-paving appearance, and may progress to diffuse alveolar damage/acute respiratory distress syndrome in severe cases [32]. In patients with diabetes, the overall imaging pattern is generally similar, whereas studies comparing diabetic and non-diabetic patients suggest a greater extent of pulmonary involvement, particularly a higher consolidation burden, in the diabetic group [33]. Superimposed bacterial infection may be considered when there is rapidly increasing consolidation with lobar predominance, pleural effusion, or cavitation. In addition to COVID-19, diabetes is also clinically associated with increased severity of other pulmonary infections, including community-acquired bacterial pneumonia, tuberculosis, and opportunistic fungal infections, which may mimic or coexist with viral pneumonia [34]. Awareness of these patterns is essential for timely diagnosis, triage, and multidisciplinary management.

### Breast

#### Diabetic mastopathy (Fig. 9)

Diabetic mastopathy is a rare, benign, fibroinflammatory condition of the breast that predominantly affects premenopausal women with long-standing type 1 or type 2 DM [35]. It occurs in 0.6–13% of patients with diabetes. It is reportedly associated with chronic hyperglycemia-induced immune dysregulation and autoimmune mechanisms, and may occur in patients with other diabetic complications [35]. Clinically, it often presents as a painless, firm breast mass (typically 0.5–6.0 cm in size), raising concerns about malignancy [35]. Most cases occur in the upper lateral/medial parts of the breast (76%) [35]. The imaging findings of diabetic mastopathy are frequently nonspecific and may closely resemble those of breast carcinoma. On ultrasound, lesions typically appear as irregular hypoechoic masses with marked posterior acoustic shadowing [35]. Mammography may demonstrate focal asymmetry or architectural distortion without microcalcifications [35]. On contrast-enhanced breast MRI, lesions often show minimal or gradual enhancement, reflecting dense fibrous tissue rather than neovascularity [35].

A major diagnostic pitfall is the misinterpretation of diabetic mastopathy as invasive breast cancer, which can lead to unnecessary aggressive surgical intervention. A clinical history of long-standing diabetes may suggest diabetic mastopathy; however, imaging alone is insufficient for a definitive diagnosis. A core needle biopsy is usually required to rule out malignancies. Recognition of this entity is crucial, as conservative management is appropriate once malignancy has been ruled out, and recurrence after surgical excision is common.

### Gastrointestinal and hepatobiliary system

#### Black esophagus

Black esophagus (acute esophageal necrosis) is a rare but life-threatening disorder characterized by circumferential esophageal mucosal necrosis (0.01–0.28%) [36], often associated with systemic hypoperfusion and diabetic ketoacidosis [36]. Because symptoms such as upper gastrointestinal bleeding or abdominal pain can overlap with those of ketoacidosis, diagnosis may be delayed; endoscopy is the diagnostic standard, showing diffuse black discoloration of the distal esophageal mucosa. CT has a supportive role and may show circumferential wall thickening with mural edema and decreased mucosal enhancement, while being most useful for evaluating complications (perforation, mediastinitis, pneumomediastinum, pleural effusion), which should prompt urgent endoscopic correlation [36].

#### Metabolic dysfunction-associated steatohepatitis and metabolic dysfunction-associated steatotic liver disease (MASH/MASLD) and fatty infiltration of the pancreas

MASH/MASLD is a common hepatic manifestation of insulin resistance and metabolic syndrome and is highly prevalent among patients with type 2 DM [4, 37]. Chronic hyperglycemia and dyslipidemia promote excessive fat accumulation within hepatocytes, causing resistance to insulin action and hepatic necro-inflammation with the activation of hepatic stellate cells and increased production of collagen matrix, as well as progression of liver disease [4]. In addition, MASH/MASLD is a major risk factor for hepatocellular carcinoma, and HCC may develop even in the absence of overt cirrhosis [4]. Imaging findings on ultrasound, CT, and MRI include increased hepatic echogenicity, reduced hepatic attenuation relative to the spleen on CT, and signal loss on opposed-phase MRI [38].

Fatty infiltration of the pancreas has also been increasingly recognized as a metabolic manifestation associated with diabetes and obesity [39]. Fatty infiltration of the pancreas appears hyperechoic on ultrasound. CT typically demonstrates diffusely decreased pancreatic attenuation, whereas MRI shows signal loss on opposed-phase imaging, reflecting fatty replacement rather than true pancreatic atrophy or tumor [40]. Fatty infiltration of the pancreas may also be observed in congenital disorders such as cystic fibrosis, Shwachman–Diamond syndrome, and Johanson–Blizzard syndrome; therefore, evaluation of the patient’s medical and family history is important [39]. Several studies have reported an association between pancreatic fatty infiltration and the development of pancreatitis and pancreatic cancer [39].

#### Xanthogranulomatous cholecystitis (XGC) (Fig. 10)

XGC is an uncommon inflammatory disease characterized by foamy macrophage infiltration of the gallbladder wall. It is frequently associated with gallstones and chronic inflammation, and histopathologically it comprises xanthogranuloma combined with foamy histiocytes, multinucleated foreign body giant cells, lymphocytes, and fibroblasts. Studies indicate that patients with diabetes are at a higher risk of developing acute cholecystitis.

Contrast-enhanced CT typically demonstrates diffuse gallbladder wall thickening with intramural nodules and mucosal line preservation [41].

A major diagnostic pitfall is confusion with gallbladder carcinoma. Diffuse gallbladder wall thickening, mucosal line preservation, presence of intramural hypo-attenuated nodules, and absence of macroscopic hepatic invasion or intrahepatic bile duct dilatation suggest XGC [42]. Furthermore, dual-energy CT appears promising for differentiating XGC from malignant disease [43].

#### Emphysematous cholecystitis (Fig. 11)

Emphysematous cholecystitis is a fulminant form of acute cholecystitis caused by gas-forming organisms and occurs predominantly in older patients with diabetes due to micro- and macro-vascular complications and neuropathic changes [44]. Gas is produced when ischemia and tissue necrosis create a hypoxic milieu that favors these organisms, and bacterial fermentation of glucose and other substrates generates gases (mainly hydrogen and carbon dioxide) that accumulate within the gallbladder wall and/or lumen [12]. This process may be facilitated by diabetes-related microvascular ischemia and elevated tissue glucose availability [44]. Rapid disease progression and a high risk of perforation make early diagnosis essential [12, 45].

Notably, a less frequently observed finding is the appearance of small, non-shadowing echogenic foci arising from the dependent portion of the gallbladder lumen on ultrasonography (*the champagne sign*). CT is the imaging modality of choice; it demonstrates gas within the gallbladder wall and lumen, with or without pericholecystic inflammation. In advanced cases, the gas may extend into the biliary tree or surrounding tissues [45]. A key pitfall is failure to distinguish between emphysematous and uncomplicated acute cholecystitis. It is easier to detect emphysema through the air window [12, 45]. Differential diagnoses include iatrogenic air and fistula formation in other organs. Confirming whether a history of medical procedures, such as drain insertion, exists or the presence of fistulas in other organs, is necessary.

### Genitourinary system

#### Xanthogranulomatous pyelonephritis (XGP) (Fig. 12)

XGP is a rare and severe form of chronic renal infection characterized by destructive granulomatous inflammation of the renal parenchyma [46]. The most commonly identified organisms are *Proteus mirabilis* and *Escherichia coli* [46]. It is frequently associated with long-standing urinary tract obstruction, nephrolithiasis (often staghorn calculus), and DM, which predispose patients to persistent infection and impaired host defense. Clinically, it is common in middle-aged to older women, and patients may present with nonspecific symptoms, including fever, flank pain, and weight loss.

Contrast-enhanced CT is the imaging modality of choice and typically demonstrates an enlarged kidney with multiple low-attenuation areas representing dilated calyces and inflammatory tissue [47], producing the characteristic “bear’s paw sign.” Perinephric fat stranding and extension into adjacent tissues may also be observed.

A major diagnostic pitfall is its misinterpretation as a renal malignancy, particularly renal cell carcinoma, because both entities may present as renal masses with invasive features. However, the presence of obstructing calculi, diffuse renal involvement, and the bear’s paw appearance favors XGP. Awareness of this entity is essential, as management usually involves nephrectomy rather than oncological resection.

#### Emphysematous pyelonephritis (EPN) and emphysematous cystitis (Fig. 13)

EPN is a life-threatening necrotizing infection caused by gas-forming organisms. It occurs predominantly in patients with systemic risk factors (including poorly controlled DM, urinary tract calculus, chronic kidney disease, malignancy, and immunocompromised status) [12, 46]. In DM, hyperglycemia and tissue ischemia promote gas production, leading to rapid disease progression and high mortality if untreated [2, 6]. The most commonly identified organisms are *Proteus mirabilis*,* Escherichia coli*, and *Klebsiella pneumonia* [46]. The symptoms may be nonspecific; however, fever, flank pain, dysuria, and nausea are common [12].

CT is crucial in diagnosis, demonstrating mottled intrarenal air lucencies with or without accompanying renal abscesses and extrarenal extension [12, 46]. The extent and distribution of these gases are critical for disease classification and prognostic assessment. The Huang–Tseng classification is a clinicoradiological system used to guide management. It categorizes EPN as follows [6]:

Class 1: gas confined to the renal collecting system;

Class 2: gas in the renal parenchyma without extension into the extrarenal space;

Class 3A: extension of gas or abscess into the perinephric space;

Class 3B: extension of gas or abscess into the pararenal space; and.

Class 4: bilateral EPN or EPN in a solitary kidney.

A key diagnostic pitfall is the underestimation of disease severity, particularly when gas is limited during the early stages. Small amounts of intraparenchymal gas should not be dismissed as benign. Differentiation from emphysematous pyelitis, in which the gas is confined to the collecting system, is essential because its management and prognosis differ substantially [12]. Differential diagnoses include iatrogenic air and fistula formation in other organs. Confirming whether a history of medical procedures, such as drain insertion, exists or the presence of fistulas in other organs, is necessary.

Emphysematous cystitis refers to an acute cystitis characterized by the presence of gas within the urinary bladder wall, with or without gas in the bladder lumen, and associated perivesical inflammation [12, 45]. Predisposing factors include DM, urinary catheterization, neurogenic bladder, urinary retention, recurrent urinary tract infections, immunosuppression, and chemotherapy [48]. The most common causative organisms are *Escherichia coli* and *Klebsiella pneumoniae* [12, 48]. Emphysematous cystitis appears as mottled lucencies outlining the bladder wall [12]. CT allows the accurate localization of gas within the bladder wall and facilitates the assessment of associated complications [12]. The prognosis of emphysematous cystitis is generally better than that of EPN [48].

#### Diabetic kidney disease (Fig. 14)

Diabetic kidney disease is a microvascular complication of DM caused by chronic hyperglycemia-induced endothelial injury, leading to glomerular basement membrane thickening, mesangial expansion, and progressive nephron loss [49]. Clinically, it is characterized by impaired renal function and/or increased urinary albumin excretion and occurs in approximately half of the patients with type 2 diabetes [49]. Imaging findings depend on the disease stage. In the early stages, the kidneys may appear normal [50]. With disease progression, ultrasonography typically shows mildly enlarged kidneys with increased cortical echogenicity and elevated resistive and pulsatility indices on Doppler imaging [50, 51]. End-stage disease is characterized by bilateral renal atrophy. Blood glucose measurements are recommended when bilateral kidney enlargement is observed for unknown reasons.

### Reproductive organs

#### Polycystic ovary syndrome (PCOS) (combined figure) (Fig. 15)

PCOS is a common endocrine disorder strongly associated with insulin resistance and hyperinsulinemia, and represents an important reproductive manifestation of metabolic dysfunction in patients with diabetes [52]. Chronic insulin resistance exacerbates excess androgen levels and anovulation, leading to characteristic ovarian and endometrial changes [52].

On ultrasound and MRI, the ovaries typically demonstrate increased volume with multiple peripherally arranged small follicles and prominent central stroma in polycystic ovarian morphology (PCOM) [53]. Follicle number per ovary (should include any follicles measuring 2–9 mm) ≥ 20, ovarian volume ≥ 10 mL, or follicle number per section ≥ 10 in at least one ovary should be considered the threshold for PCOM in adults [53]. In addition, prolonged unopposed estrogen exposure predisposes patients to endometrial hyperplasia and increases the risk of endometrial carcinoma [54].

A diagnostic pitfall is the overreliance on ovarian morphology alone, as polycystic-appearing ovaries may be observed in asymptomatic women. Imaging findings must be interpreted in conjunction with clinical and biochemical criteria. Careful evaluation of the endometrial thickness and signal characteristics is essential to avoid missing clinically significant endometrial pathologies.

#### Vas deferens calcification (combined figure) (Fig. 15)

Vas deferens calcification is an uncommon but characteristic imaging finding that is frequently observed in patients with long-standing DM [55]. Chronic hyperglycemia and hyperphosphatemia due to insulin resistance are thought to promote medial calcification of the vas deferens, often detected incidentally on CT scans performed for unrelated indications [55]. Imaging typically reveals linear or tubular calcifications along the expected course of the vas deferens, usually bilateral and symmetric [56]. Calcifications occur within the muscular components, with preservation of luminal patency [56]. These calcifications are generally asymptomatic but may be associated with infertility in some cases. Chronic inflammatory calcification is intraluminal, often unilateral, and segmental [56]. Recognition of the anatomical course and characteristic morphology of vas deferens calcifications helps prevent diagnostic confusion and unnecessary further evaluation.

### Musculoskeletal and soft tissue

#### Necrotizing fasciitis and Fournier gangrene (combined figure) (Fig. 16)

Necrotizing fasciitis is a rapidly progressive, life-threatening soft-tissue infection that predominantly involves the deep fascia [7, 57]. CT is the primary imaging modality in acute settings because of its speed and sensitivity for detecting gas [57]. Typical CT findings include linear or loculated gas tracking along the fascial planes, diffuse fascial thickening and enhancement, and deep soft-tissue edema extending beyond the area of skin involvement [57]. MRI provides superior soft-tissue contrast and may demonstrate thick (≥ 3 mm) abnormal signal intensity on fat-suppressed T2-weighted images, low signal intensity in the deep fascia on fat-suppressed T2-weighted images, a focal or diffuse nonenhancing portion in the area of abnormal signal intensity in the deep fascia, extensive involvement of the deep fascia, and involvement of three or more compartments in one extremity [58].

Fournier gangrene is a rapidly progressive, life-threatening, necrotizing infection of the perineal and genital soft tissues that predominantly occurs in patients with DM [8]. Hyperglycemia-related immune dysfunction, microvascular disease, and persistent hyperglycemia facilitate rapid bacterial proliferation and tissue ischemia, predisposing patients to extensive necrosis [2]. SGLT-2 inhibitors increase the risk of infection due to urinary glucose in Fournier gangrene [59]. Necrotizing fasciitis may coexist or extend beyond the perineum, involving deeper fascial planes in Fournier gangrene.

CT is the imaging modality of choice and typically demonstrates subcutaneous emphysema, fascial thickening, fluid collection, and the loss of normal tissue planes [8]. Extension along the fascial plane is a key imaging feature distinguishing necrotizing infections from superficial cellulitis.

A critical diagnostic pitfall is the underestimation of disease extent, particularly when gas is minimal or absent in the early stages. The absence of gas does not exclude necrotizing fasciitis [8]. Prompt recognition of subtle fascial edema and asymmetric soft-tissue involvement is essential, as delayed diagnosis significantly increases mortality [8]. Imaging should not delay urgent surgical intervention when clinical suspicion is high [8].

#### Osteomyelitis (Fig. 17)

Osteomyelitis in patients with DM most commonly results from a combination of peripheral neuropathy, vascular insufficiency, and impaired immune response. They frequently involve the foot and ankle and may arise from the contiguous spread of soft-tissue infections, particularly in the setting of diabetic foot ulcers [7]. Ulcers tend to occur at anatomic sites that are subjected to the highest contact pressures during ambulation, and large (> 2 cm) and deep (> 3 mm) ulcers are more likely to be associated with infections of the underlying bone [7]. Early diagnosis is crucial because delayed treatment can lead to irreversible bone destruction and limb-threatening complications.

MRI is the imaging modality of choice for suspected osteomyelitis, demonstrating bone marrow edema with low signal intensity on T1-weighted images and high signal intensity on fluid-sensitive sequences, often accompanied by adjacent soft-tissue inflammation or abscess formation. Contrast-enhanced MRI may further delineate devitalized bone and sinus tracts. CT is useful for detecting cortical destruction, sequestra, and gas, particularly in chronic cases [7].

#### Charcot arthropathy (Fig. 18)

Charcot arthropathy is a destructive osteoarthropathy that most commonly affects the foot and ankle in patients with diabetic peripheral neuropathy [60]. Early recognition is crucial because prompt off-loading/immobilization can prevent progressive deformity. In the acute (active) stage, MRI is the most sensitive modality and may show periarticular/subchondral bone marrow edema, microfractures, joint effusion, and soft-tissue edema even when radiographs are normal. With progression, radiographs/CT demonstrate fragmentation, subluxation/dislocation, osseous debris, collapse of the midfoot architecture, and subsequent remodeling, sometimes resulting in a rocker-bottom deformity [60]. A key diagnostic challenge is its differentiation from osteomyelitis in diabetic feet; osteomyelitis more often shows focal marrow abnormality contiguous with an ulcer/sinus tract and may be accompanied by abscess, whereas Charcot changes are typically more periarticular with a predominant midfoot distribution [60].

#### Diffuse idiopathic skeletal hyperostosis (DISH) (Fig. 19)

DISH is characterized by exuberant ossification at sites of tendinous and ligamentous insertion (entheses) and is associated with metabolic conditions, including DM [61]. Although often asymptomatic, it may cause back pain and spinal stiffness. On radiography or CT, DISH shows flowing anterior ossification bridging at least four contiguous vertebral bodies with preserved disc height, and no sacroiliac erosions/sclerosis/fusion; facet and costovertebral joint ankylosis is typically absent, helping distinguish DISH from inflammatory spondyloarthropathies. It commonly predominates in the right thoracic spine. Identification is essential because ankylosed segments are prone to unstable fractures after minor trauma; CT is useful for fracture detection, and MRI can assess marrow edema and soft-tissue injury when needed.

#### Hyperostosis frontalis interna (HFI) (Fig. 20)

HFI is a benign, typically incidental hyperostotic change characterized by symmetric, smooth thickening of the inner table of the frontal bone, with preservation of the outer table and diploic space. It is most often seen in older women and may coexist with metabolic disorders, including long-standing diabetes [62]. Recognition of this characteristic distribution helps avoid confusion with other hyperostotic conditions such as Paget’s disease (involvement of both inner and outer tables with trabecular coarsening) [63] or focal hyperostosis adjacent to an extra-axial mass (e.g., en plaque meningioma). No specific treatment is required.

### Treatment-related imaging findings

#### Pneumatosis intestinalis (Fig. 21)

Pneumatosis intestinalis is characterized by gas-filled cysts within the bowel wall [64] and may be associated with DM, particularly in patients receiving alpha-glucosidase inhibitors [65]. This condition ranges from benign to life-threatening depending on the underlying cause [64, 66]. CT demonstrates multiple cystic or linear collections of gas within the bowel walls. In benign forms, bowel wall enhancement is preserved, and no portal venous gas or pneumoperitoneum is observed [66]. A significant diagnostic pitfall is the overdiagnosis of bowel ischemia. The absence of reduced mural enhancement, mesenteric vessel occlusion, or severe clinical symptoms indicates a benign etiology, allowing conservative management in selected patients.

#### Metformin-related bowel FDG uptake (Fig. 22)

Metformin causes increased physiological FDG uptake in the bowel on PET/CT owing to enhanced glucose transport and metabolism in the intestinal mucosa [11, 67]. FDG-PET/CT typically demonstrates diffuse, intense tracer uptake throughout the colon and small bowel [11]. A common pitfall is the misinterpretation as inflammatory bowel disease or malignancy. Awareness of metformin use and, when necessary, temporary drug discontinuation before imaging can prevent false-positive interpretations and unnecessary further investigations.

#### Insulin ball (Fig. 23)

An insulin ball is a localized, subcutaneous amyloid deposit caused by repeated insulin injections at the same site [68]. An insulin ball is a firm, palpable, subcutaneous mass showing heterogeneous attenuation on CT and low signal intensity on both T1- and T2-weighted MRI [69], with little or no change after the cessation of insulin injections.

A key diagnostic pitfall is the misinterpretation of an insulin ball as lipohypertrophy [70]. Chronic mechanical stimulation and anabolic effects of insulin promote localized adipose tissue hypertrophy, resulting in palpable subcutaneous masses. Lipohypertrophy presents as a soft lesion with CT attenuation and MRI signal intensity identical to normal fat and typically demonstrates rapid shrinkage after discontinuing repeated insulin injections. These lesions are typically located at common injection sites, such as the abdominal wall or thigh.

## Clinical implications for radiologists

Radiologists play an important role in the early detection and management of diabetes-related complications. Given the multisystem nature of DM, accurate interpretation requires a comprehensive and context-aware approach.

From a radiological perspective, the detection of diabetic complications before symptom onset is crucial. Imaging can reveal hepatic steatosis, pancreatic fat infiltration, cerebral white matter lesions, subclinical CAD, and renal morphological changes, thereby enabling early risk stratification and intervention.

Early recognition of life-threatening conditions—such as malignant otitis externa, emphysematous infections, necrotizing fasciitis, and Fournier gangrene—is also critical. CT findings, including soft tissue or parenchymal gas, deep fascial fluid, and asymmetric inflammatory changes, should prompt urgent multidisciplinary intervention.

Radiologists should also be aware of metabolic and medication-related imaging pitfalls, such as diffuse bowel FDG uptake associated with metformin use and an insulin ball. Familiarity with benign diabetic conditions that mimic malignancies, including diabetic mastopathy and xanthogranulomatous inflammation, helps prevent unnecessary invasive procedures.

In addition, diabetes and insulin resistance have been associated with an increased risk of several malignancies (e.g., hepatocellular carcinoma, pancreatic, colorectal, breast, and endometrial cancers), potentially through hyperinsulinemia/insulin-like growth factor signaling and chronic low-grade inflammation, with MASLD/MASH serving as an important intermediary pathway, particularly for hepatocellular carcinoma [4]. However, the imaging features of these cancers are generally not diabetes-specific; therefore, radiologists should prioritize appropriate cancer surveillance in high-risk patients and avoid over-attributing nonspecific imaging findings to diabetes alone.

By integrating imaging findings with clinical and metabolic contexts, radiologists can contribute to accurate diagnosis, early treatment, and optimized multidisciplinary care.

**Fig. 1:**
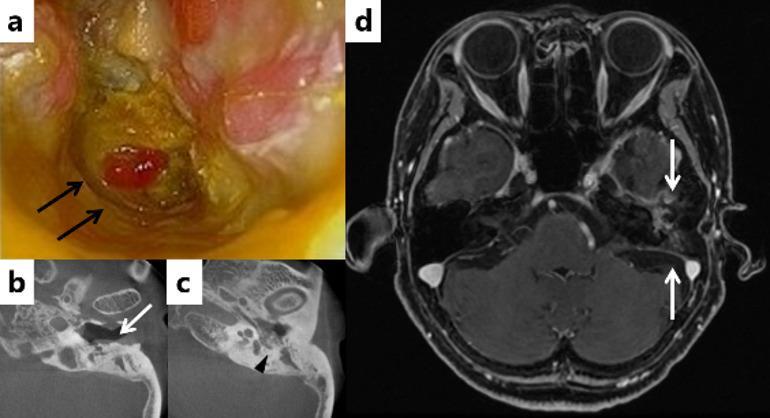
Malignant otitis externa. **a** Otoscopy, **b**–**c** CT, **d** contrast-enhanced MRI. A woman in her 70s with type 2 DM presented with left ear leakage and pain. Otoscopy (**a**) reveals the exposed bone in the wall of the external auditory canal (arrow). Axial CT (**b** and **c**) shows soft-tissue thickening of the external auditory canal and tympanic cavity (arrow) with focal erosion of the temporal bone (arrowhead). Contrast-enhanced MRI (**d**) demonstrating marrow edema and enhancement extending toward the skull base. These findings reflect invasive infections in immunocompromised patients with diabetes and warrant urgent treatment. Abbreviation: CT, computed tomography; MRI, magnetic resonance imaging; DM, diabetes mellitus

**Fig. 2:**
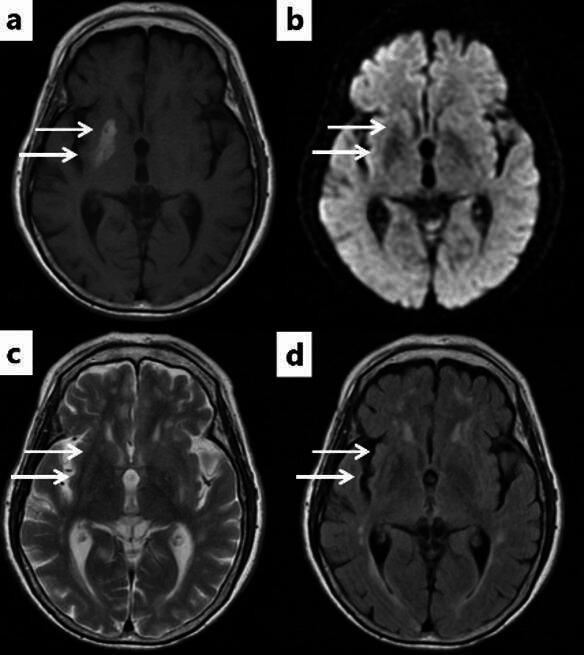
Non-ketotic hyperglycemic hemichorea. **a** T1WI, **b** DWI, **c** T2WI, **d** FLAIR. A woman in her 70s with type 2 DM presented with new-onset left arm and leg numbness. Axial T1WI (**a**) reveals marked unilateral hyperintensity in the basal ganglia (arrow) contralateral to the involuntary movements. Abbreviations: DM, diabetes mellitus; FLAIR, fluid-attenuated inversion recovery; DWI, diffusion-weighted imaging

**Fig. 3:**
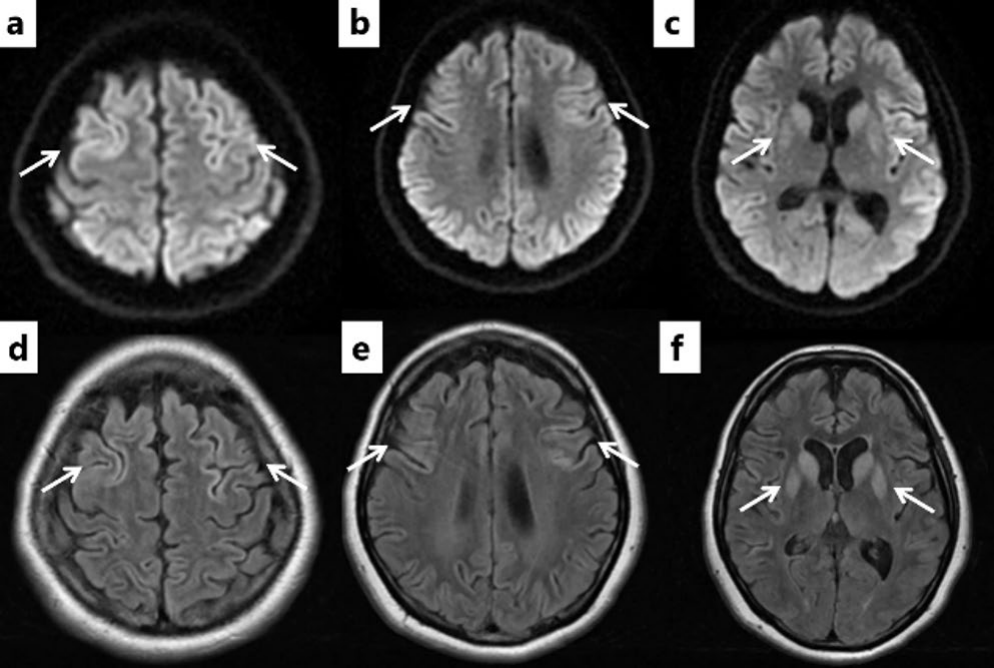
Hypoglycemic encephalopathy. **a**–**c** DWI, **d**–**f** FLAIR. A woman in her 30s with type 2 DM accidentally ingested a large sulfonylurea dose, resulting in prolonged loss of consciousness. DWI (**a**–**c**) and FLAIR (**d**–**f**) show bilateral symmetric hyperintensities in the frontal and parietal lobes and the striatum (arrow). This distribution differs from that of vascular territorial infarctions. Abbreviations: DM, diabetes mellitus; FLAIR, fluid-attenuated inversion recovery; DWI, diffusion-weighted imaging

**Fig. 4:**
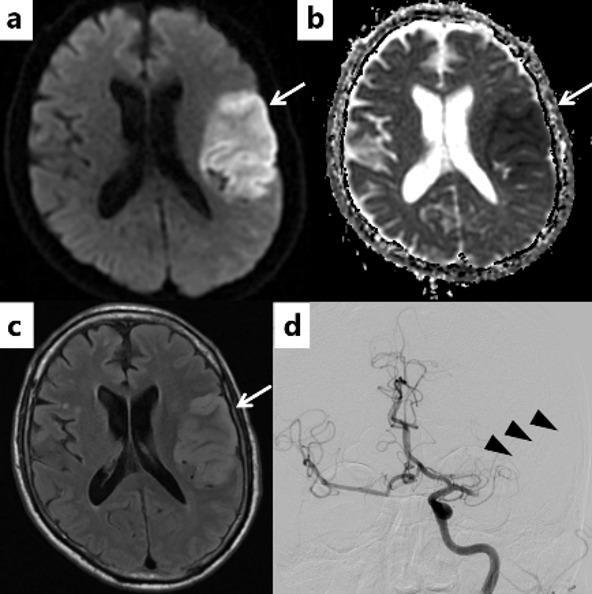
Stroke. **a** DWI, **b** ADC map, **c** FLAIR, **d** DSA.A man in his 50s with type 2 DM had sudden-onset right-sided hemiplegia. MRI (**a**–**c**) reveals a large left MCA territory infarct (arrow). DSA reveals left MCA obstruction (arrowhead). Intra-arterial thrombolysis resulted in complete left MCA recanalization. Abbreviations: DM, diabetes mellitus; FLAIR, fluid-attenuated inversion recovery; MCA, middle cerebral artery; DSA, digital subtraction angiography; MRI, magnetic resonance imaging; DWI, diffusion-weighted imaging

**Fig. 5:**
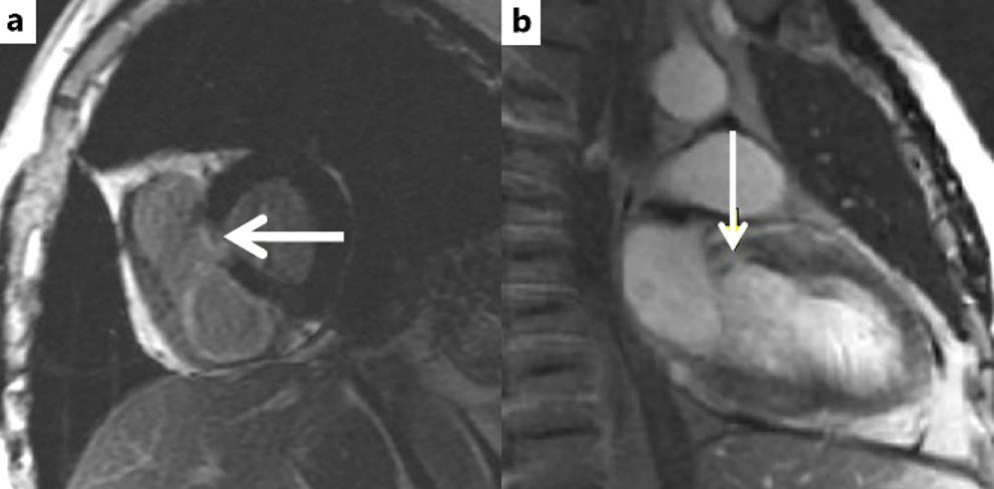
Diabetic cardiomyopathy. **a**–**b** MRI (late gadolinium enhancement; LGE). A man in his 50s with type 2 DM, whose left ventricular ejection fraction decreased to approximately 50%. Cardiac MRI **a**–**b** reveals delayed enhancement in the anterior septum at the base of the heart (arrow). The native T1 value was 1221 ms, which was within the normal range. CT Angiography shows no significant stenosis in the coronary arteries (data not shown), indicating diabetic cardiomyopathy independent of the coronary artery distribution. Abbreviations: LGE, late gadolinium enhancement; CT, computed tomography; DM, diabetes mellitus; MRI, magnetic resonance imaging

**Fig. 6:**
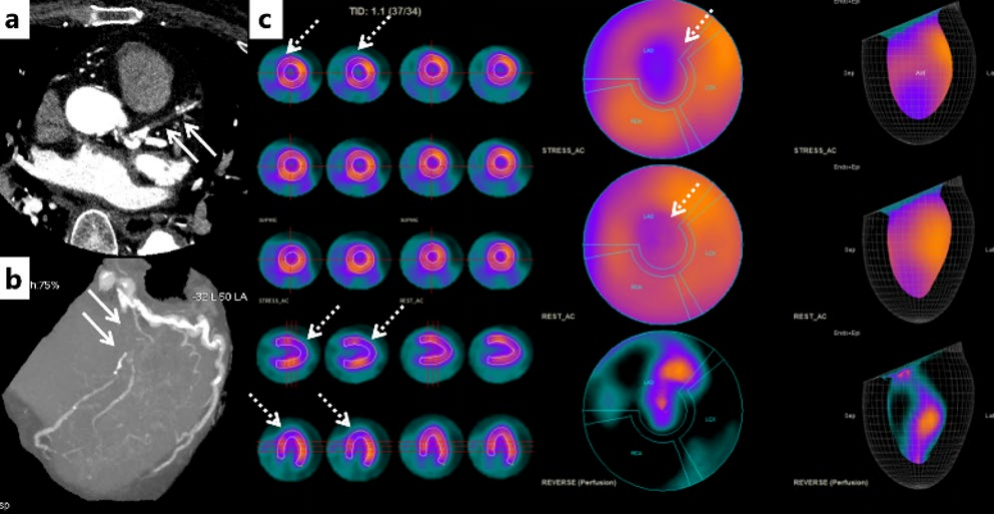
Coronary artery disease (CAD). **a** CCTA, **b** CCTA (3D reconstruction), **c** ^201^Tl myocardial perfusion SPECT. A woman in her 80s with type 2 DM presented with chest pain. CCTA (**a**–**b**) shows 99% stenosis or occlusion of the distal LAD (arrow). ^201^Tl myocardial perfusion SPECT with pharmacological stress testing (dipyridamole) reveals myocardial ischemia at the mid-to apical anterior wall (dotted arrow). Abbreviation: LAD, left anterior descending coronary artery; CCTA, coronary CT angiography; SPECT, single photon emission computed tomography

**Fig. 7:**
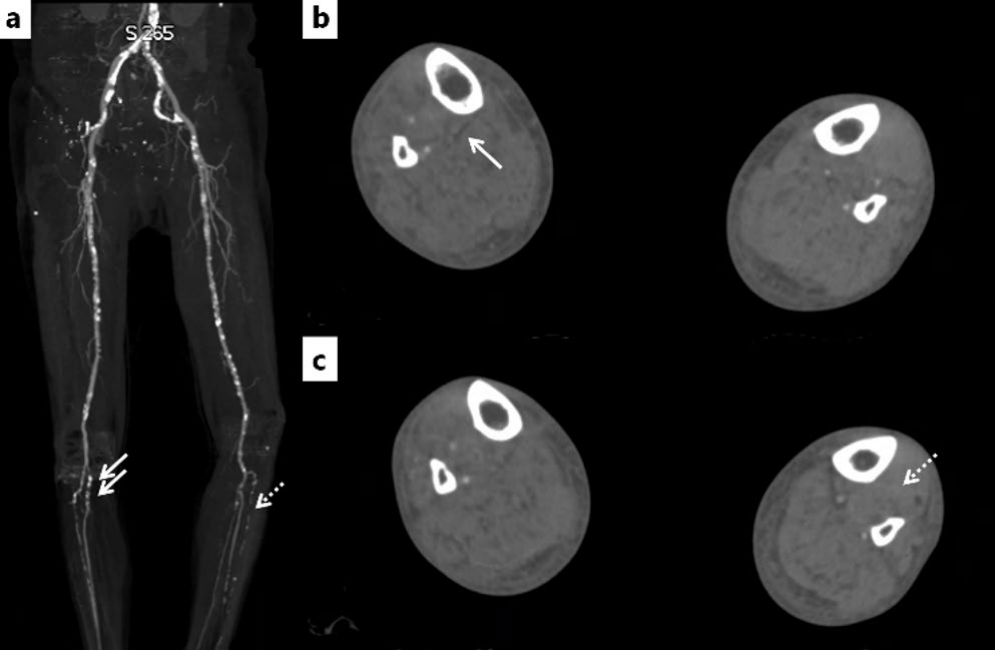
Peripheral artery disease (PAD). **a** CTA, **b**–**c** axial CT. A woman in her 70s with type 2 DM presented with numbness and pain in both lower extremities. MIP image from peripheral CTA demonstrates extensive atherosclerotic disease with multifocal stenosis/occlusion. The right posterior tibial artery is occluded (arrow) and the proximal left anterior tibial artery is occluded (dotted arrow). Abbreviations: DM, diabetes mellitus; MIP, maximum-intensity projection; CTA, computed tomography angiography

**Fig. 8:**
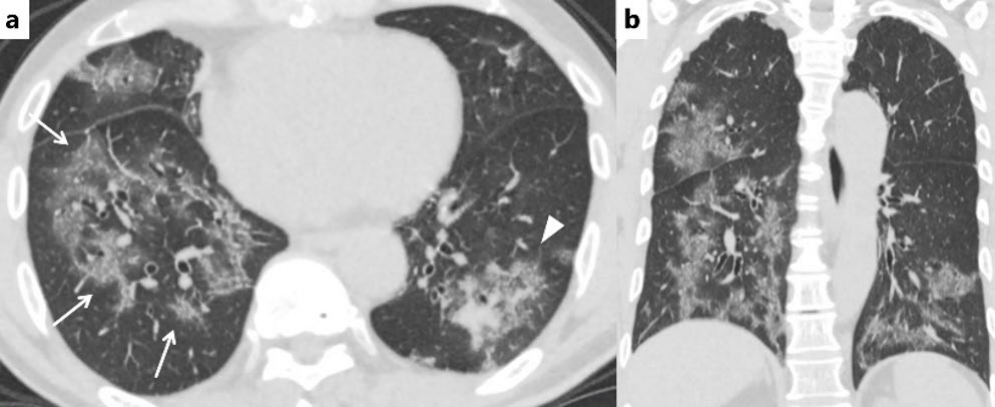
Coronavirus disease 2019 (COVID-19). **a** Axial CT, **b** coronal CT. A man in his 70s with type 2 DM presented with several days of fever, cough, fatigue, and exertional dyspnea. Chest CT demonstrates multiple bilateral, peripheral-predominant ground-glass opacities (arrows) and a consolidation in the left lower lobe (arrowhead). The diagnosis of COVID-19 pneumonia was confirmed by PCR testing. Abbreviations: CT, computed tomography; DM, diabetes mellitus; GGO, ground-glass opacity; PCR, polymerase chain reaction

**Fig. 9:**
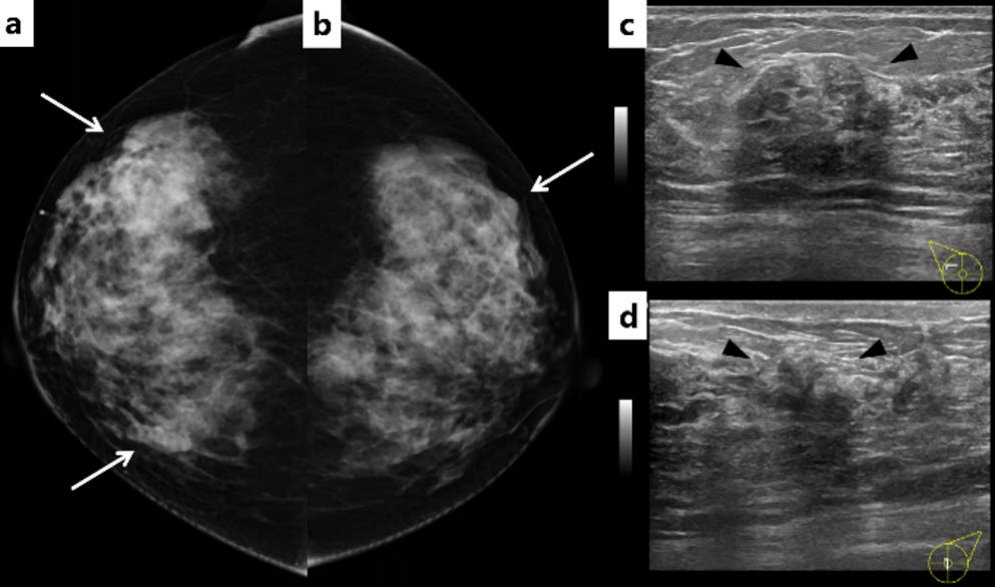
Diabetic mastopathy. **a**–**b** Mammography, **c**–**d** ultrasound. A woman in her 60s with type 2 DM underwent dialysis for diabetic kidney disease. Mammography (**a**–**b**) shows bilateral asymmetric local densities (arrows). Ultrasound (**c**–**d**) shows hypoechoic lesions with posterior acoustic shadowing (arrowhead). Biopsy revealed diabetic mastopathy, and ultrasound and mammography showed no gross interval changes in the imaging findings over time (not shown). Abbreviation: DM, diabetes mellitus

**Fig. 10:**
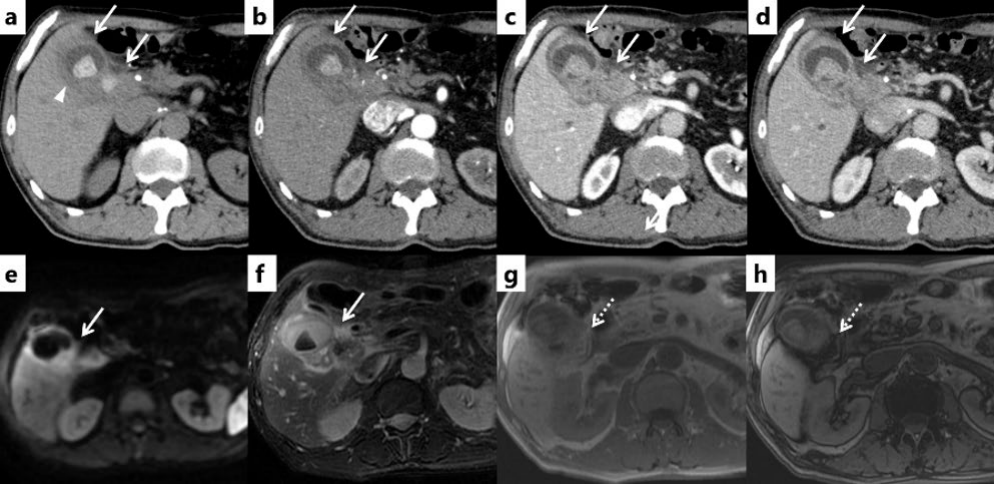
Xanthogranulomatous cholecystitis (XGC). **a**–**d** Dynamic contrast-enhanced CT: **a** Plain CT, **b** arterial phase, **c** portal phase, **d** equilibrium phase, **e**–**h** MRI: **e** DWI, **f** fat-suppressed T2WI, **g**–**h** chemical shift imaging: **g** in phase, **h** opposed phase. A man in his 70s with type 2 DM underwent CT evaluation for elevated hepatobiliary enzyme levels and chronic abdominal pain. Contrast-enhanced CT (**a**–**d**) showing gallstones (a; arrowhead) and diffuse thickening of the gallbladder wall (**a**–**d**; arrow). MRI shows diffusion restriction in the wall of the gallbladder on DWI (**e**; arrow). Chemical shift imaging showing low fat intensity (**g**–**h**; dotted arrow). A cholecystectomy was performed, and the patient was diagnosed with XGC. Abbreviations: XGC, xanthogranulomatous cholecystitis; CT, computed tomography; MRI, magnetic resonance imaging; DWI, diffusion-weighted imaging; T2WI, T2-weighted imaging

**Fig. 11:**
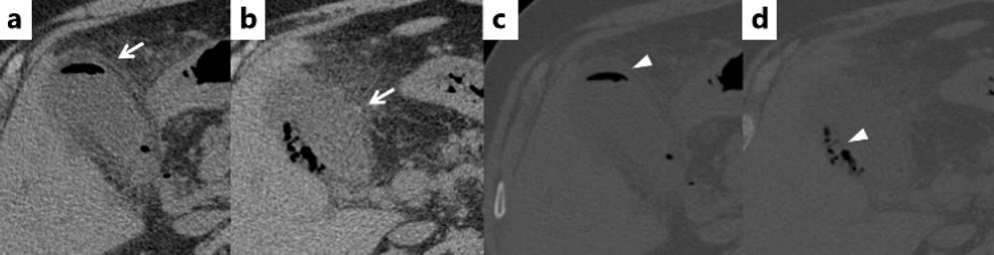
Emphysematous cholecystitis. **a**–**b** CT, **c**–**d** CT (bone window). A man in his 70s with type 2 DM presented with a chief complaint of abdominal pain. CT (**a**–**d**) demonstrates gallbladder wall thickening, pericholecystic fluid, and inflammatory fat stranding (arrow). Emphysematous cholecystitis was suspected because of the presence of air along the gallbladder wall (arrowhead). Cholecystectomy was performed, and the patient was diagnosed with emphysematous cholecystitis. Abbreviations: CT, computed tomography; DM, diabetes mellitus

**Fig. 12:**
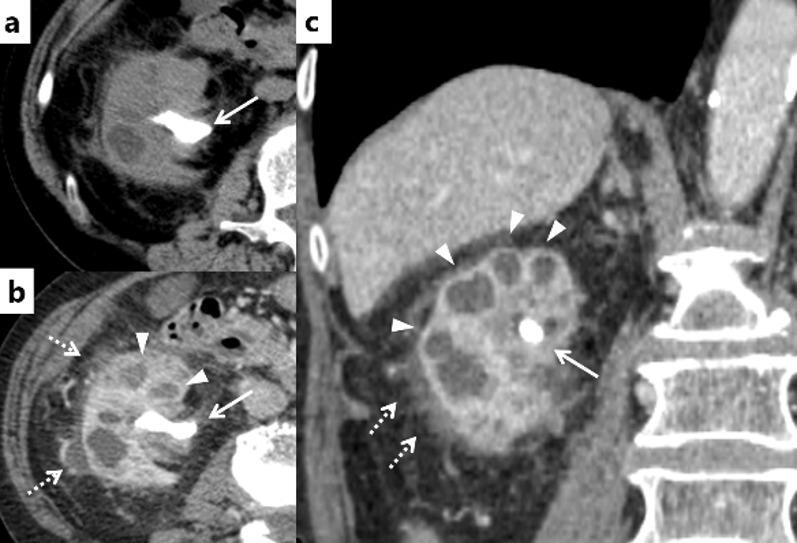
Xanthogranulomatous pyelonephritis (XGP). **a** Plain CT (axial image), **b** contrast-enhanced CT (axial image), **c** contrast-enhanced CT (coronal image). A woman in her 80s with type 2 DM had a right staghorn stone (**a**–**c**; arrow) and repeated chronic urinary tract infections. She presented with fever, pyuria, and elevated WBC and CRP. CT demonstrates the characteristic “bear’s paw sign,” with dilated calyces in the right kidney with contrast enhancement at the margins (**b**–**c**; arrowheads). Perinephric fat stranding is observed around the right kidney (**b**–**c**; dotted arrow). Right nephrectomy was performed, and the patient was diagnosed with XGP. Abbreviation: XGP, Xanthogranulomatous pyelonephritis; DM, diabetes mellitus; CT computed tomography; WBC, white blood cell; CRP, C-reactive protein

**Fig. 13:**
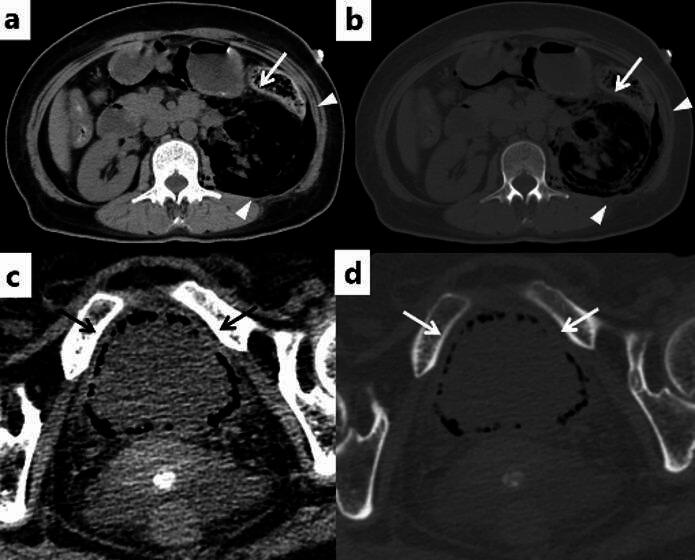
Emphysematous pyelonephritis (EPN) and emphysematous cystitis. *EPN* **a** CT, **b** CT (bone window). A woman in her 50s with type 2 DM complained of gradually worsening left abdominal pain and pyuria. CT shows left kidney enlargement (**a**–**b**; arrow) and air extending into the perirenal space (a-b; arrowhead). *Emphysematous cystitis* **c** CT, **d** CT (bone window). A woman in her 60s with type 2 DM had persistent fever, anorexia, pyuria, and hematuria. CT shows air in the bladder wall with bladder wall thickening (**c**, **d**; arrow). Abbreviations: CT, computed tomography; DM, diabetes mellitus; EPN, Emphysematous pyelonephritis

**Fig. 14:**
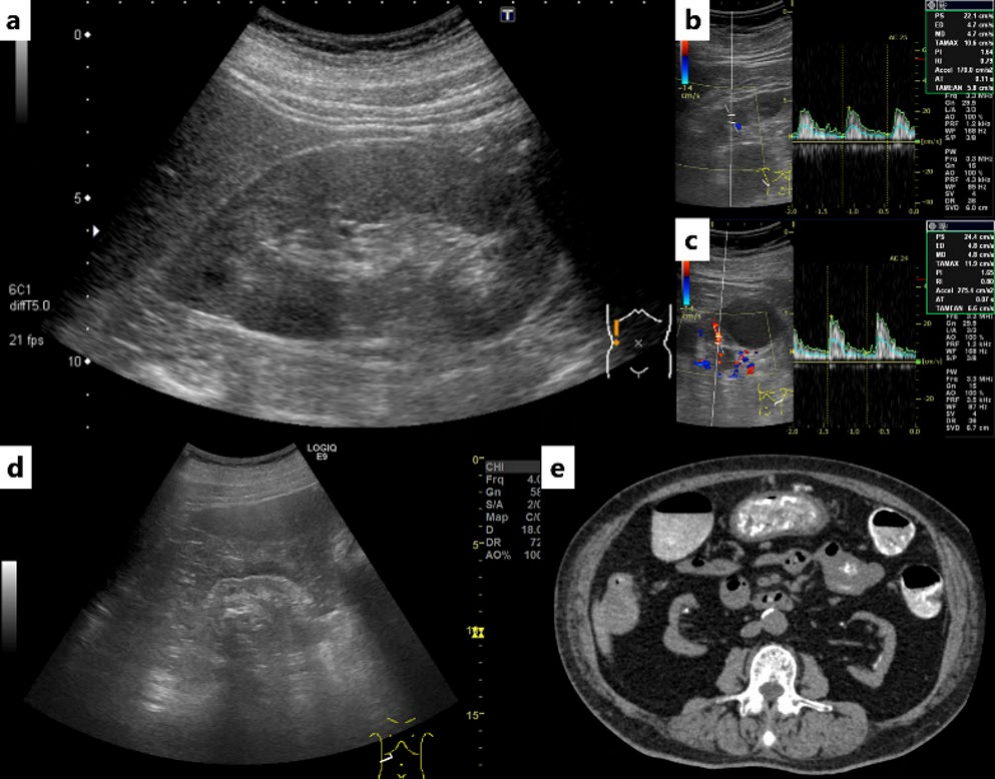
Diabetic kidney disease. *The early stage* **a**–**c** ultrasound. A man in his 70s with type 2 DM (eGFR: 19). Ultrasound shows mildly enlarged bilateral kidneys with increased cortical echogenicity (**a**). Doppler ultrasound reveals an increased RI and PI (**b**–**c**). PI values were 1.64 (right) and 1.65 (left) (normal range, 0.9–1.3), and RI values were 0.79 (right) and 0.80 (left) (normal range, ≤0.70). *The end stage* **d** ultrasound, **e** CT. A woman in her 60s with type 2 DM (eGFR: 5) undergoing pre-dialysis evaluation. Renal ultrasound demonstrates bilateral renal atrophy with diffusely increased cortical echogenicity (**d**), consistent with advanced chronic parenchymal disease. Non-contrast CT shows marked bilateral renal atrophy (**e**). Abbreviations: DM, diabetes mellitus; eGFR, estimated glomerular filtration rate; RI, resistive index; PI, pulsatility index; CT, computed tomography

**Fig. 15:**
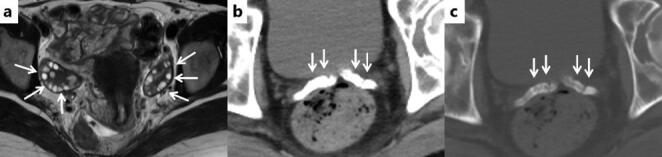
Polycystic ovary syndrome and vas deferens calcification. *PCOS* **a** T2WI. Pelvic MRI showing bilaterally enlarged ovaries with multiple small follicles arranged peripherally and increased stromal echogenicity, typical of PCOS associated with insulin resistance (**a**; arrow). *Calcification of the Vas Deferens* **b** CT, **c** CT (air window). CT demonstrates bilateral linear calcifications along the vas deferens, a characteristic and benign finding in patients with long-standing diabetes (**b**–**c**; arrow). Abbreviations: PCOS, polycystic ovary syndrome; MRI, magnetic resonance imaging; CT, computed tomography

**Fig. 16:**
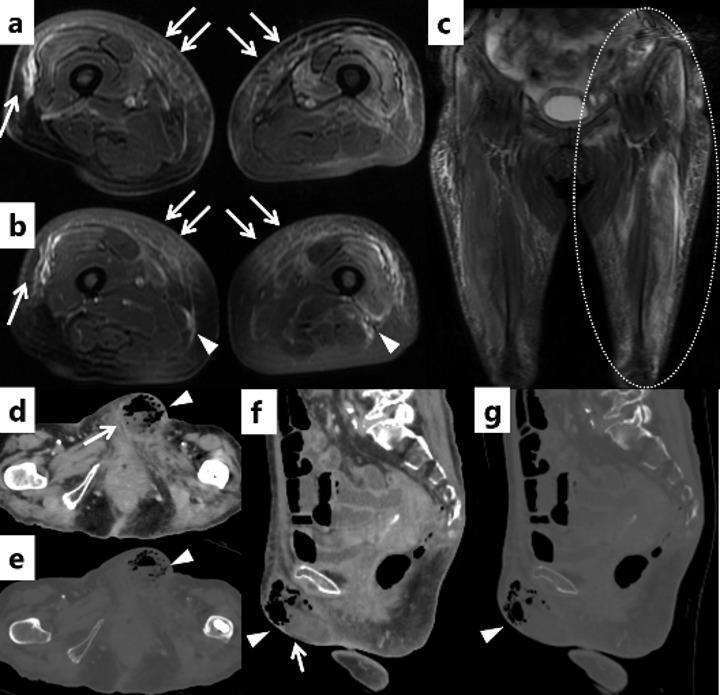
Necrotizing fasciitis and Fournier gangrene. *Necrotizing fasciitis* **a**–**c** fat-suppressed T2WI: **a**–**b** axial image and **c** coronal image. A woman in her 60s with type 2 DM experienced bilateral thigh pain. She was undergoing hemodialysis for diabetic nephropathy. High signal intensity of the subcutaneous fat layer (**a**–**b**; arrow) in the lower extremities is observed on fat-suppressed T2WI (fsT2WI). Abnormal signal intensity is also observed in the deep fascia (**a**–**b**; arrowhead) and muscles (**c**; dotted circle). *Fournier gangrene* **d** axial CT, **e** axial CT (bone window), **f** sagittal CT, **g** sagittal CT (bone window). A woman in her 80s with type 2 DM experienced left perineal pain, redness, and swelling. CT showing fat stranding, fluid (**d** and **f**; arrow), and subcutaneous gas (**d**–**g**; arrowhead) locules of soft tissue in the left perineal region. Abbreviations: CT, computed tomography; DM, diabetes mellitus

**Fig. 17:**
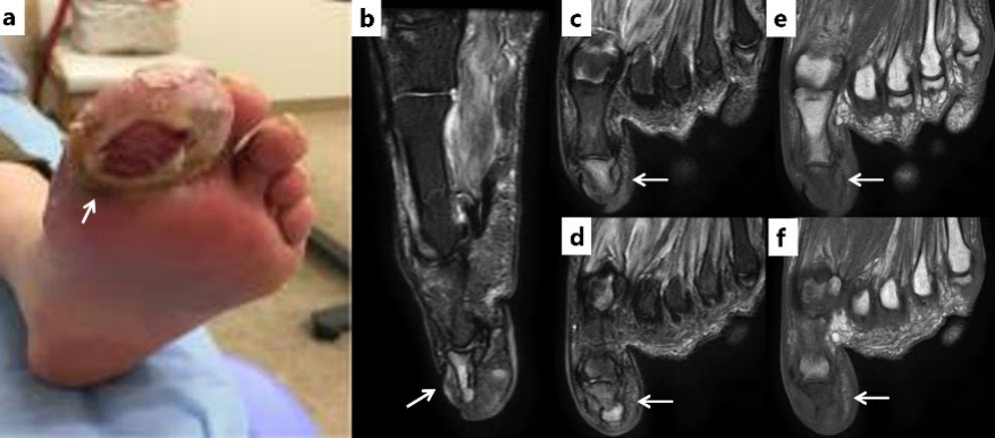
Osteomyelitis. **a** Gross appearance, **b**–**f** MRI: **b** STIR (sagittal image), **c**–**d** STIR (coronal image), **e**–**f** T1WI (coronal image). A woman in her 50s with type 2 DM presented with cellulitis of the left lower extremity and ulceration of the great toe (**a**). The bone marrow of the distal phalanx shows high signal intensity on STIR images and low signal intensity on T1WI images (**b**–**f**; arrow). Osteomyelitis caused by an ulcer was suspected, and the patient’s symptoms improved with antibiotic therapy. Abbreviations: MRI, magnetic resonance imaging; STIR, short-tau inversion recovery

**Fig. 18:**
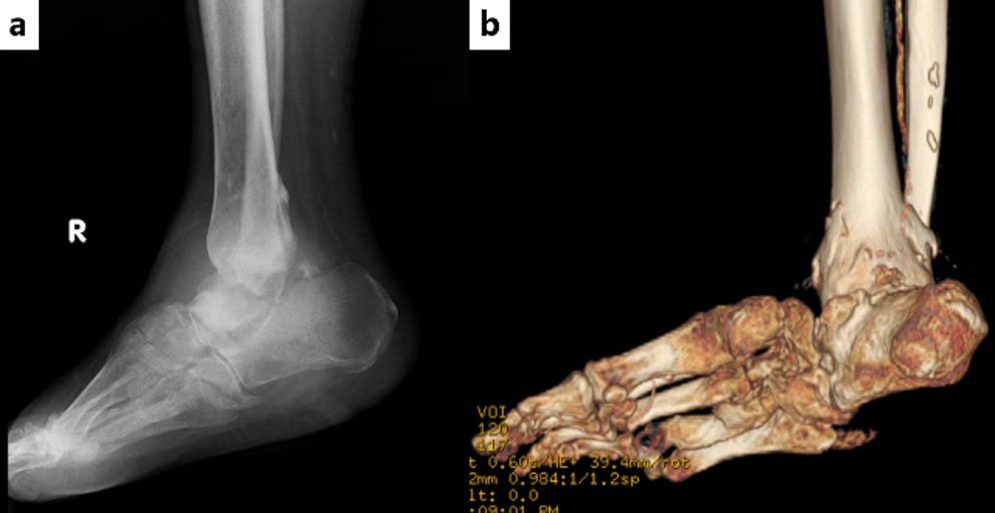
Charcot arthropathy. **a** lateral radiograph, **b** 3DCT. A woman in her 50s with type 2 DM presented with right foot deformity. Lateral radiograph (**a**) and CT (**b**) demonstrate marked deformity of the 3rd and 4th metatarsals, with multifocal periarticular osseous destruction and subchondral irregularity involving the calcaneus, navicular, cuboid, and cuneiform bones. Abbreviations: CT, computed tomography; DM, diabetes mellitus

**Fig. 19:**
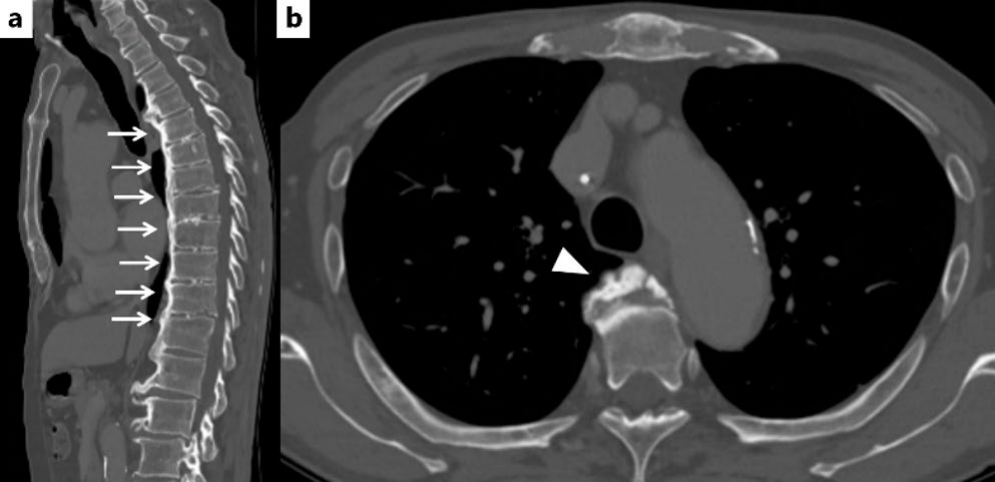
Diffuse idiopathic skeletal hyperostosis (DISH). **a** sagittal CT (bone window), **b** axial CT (bone window). A man in his 70s with asymptomatic type 2 DM. Sagittal CT (**a**) shows flowing anterior ossification bridging multiple contiguous vertebral bodies (arrow) with relative preservation of disc height. Axial CT (**b**) at the corresponding level demonstrates bulky anterolateral ossification (arrowhead). Abbreviations: CT, computed tomography; DM, diabetes mellitus

**Fig. 20:**
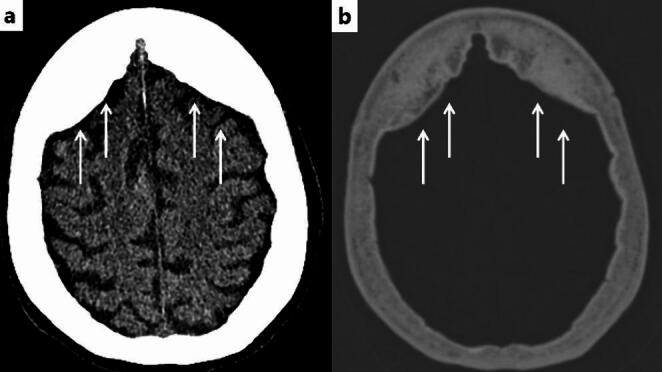
Hyperostosis frontalis interna (HFI). **a** CT, **b** CT (bone window). Non-contrast CT in the bone window demonstrated symmetric, smooth thickening of the inner table of the frontal bone with preservation of the outer table and diploic space. This benign finding is frequently associated with metabolic disorders and should not be mistaken for Paget’s disease or tumor-related hyperostosis. Abbreviation: CT, computed tomography; HFI, Hyperostosis frontalis interna

**Fig. 21:**
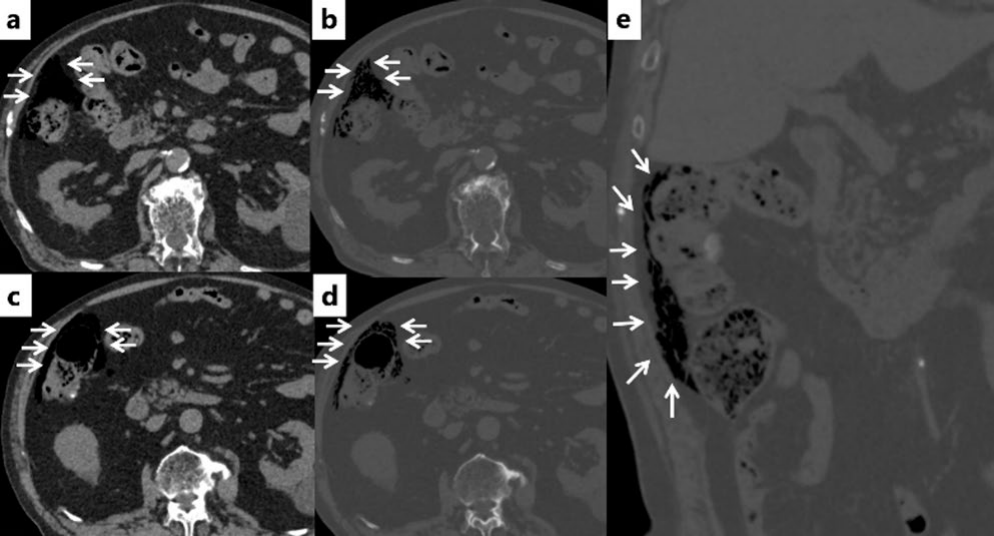
Pneumatosis cystoides intestinalis. **a** axial CT, **b** axial CT (bone window), **c** axial CT, **d** axial CT (bone window), **e** coronal CT (bone window). A man in his 80s with type 2 DM presented with no symptoms. He was taking alpha-glucosidase inhibitors to treat DM. Screening CT showed air along the colon wall (**a**–**e**; arrow) incidentally, and pneumatosis cystoides intestinalis was suspected. CT reveals multiple cystic collections of gas within the bowel wall without reduced mural enhancement or portal venous gas, suggesting a non-ischemic etiology in the appropriate clinical context (not shown). It disappeared on follow-up CT without any treatment (data not shown). Abbreviations: CT, computed tomography; DM, diabetes mellitus

**Fig. 22:**
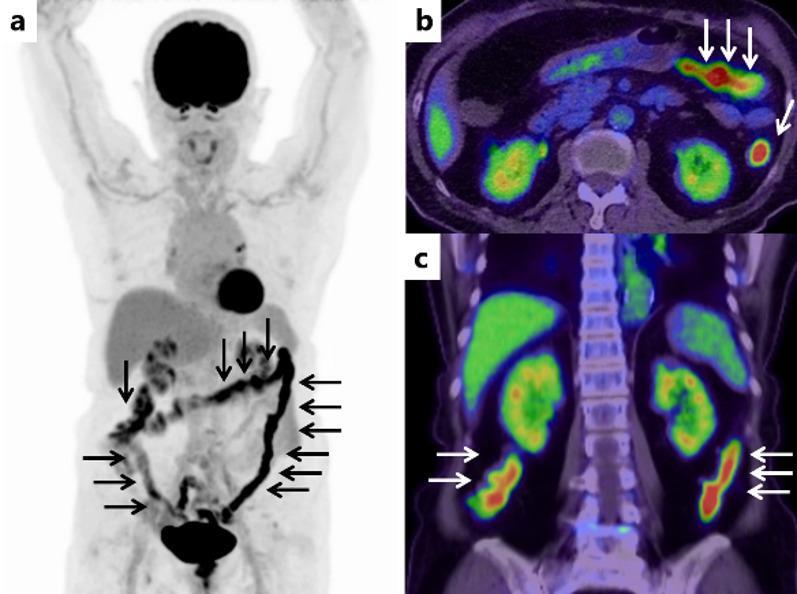
Metformin-related bowel FDG uptake. **a** FDG-PET/CT (MIP image), **b** FDG-PET/CT (fusion image) (axial image), (c) FDG-PET/CT (fusion image) (coronal image). A man in his 80s with type 2 DM treated with metformin presented without any symptoms. FDG-PET/CT showing diffuse uptake throughout the colon and distal small intestine (SUVmax: 6.8) (**a**–**c**; arrow). No bowel wall thickening or inflammation was observed in the FDG-positive bowel (data not shown). Abbreviations: FDG-PET/CT, F-fluorodeoxyglucose positron emission tomography/computed tomography; FDG, F-fluorodeoxyglucose; DM, diabetes mellitus

**Fig. 23:**
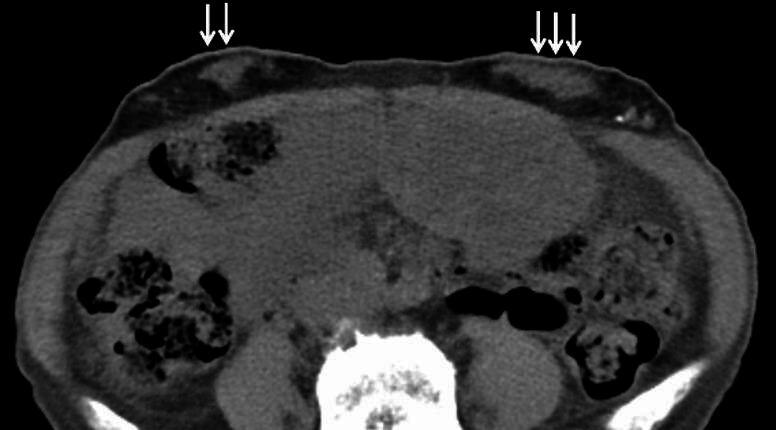
Insulin ball. A man in his 80s with asymptomatic type 2 DM had been subcutaneously injected with insulin for a long time. CT shows heterogeneous subcutaneous nodules (arrow). Abbreviations: CT, computed tomography; DM, diabetes mellitus

## Conclusion

DM is a systemic disease associated with a wide range of structural, metabolic, vascular, and infectious complications involving nearly every organ system. Understanding the underlying pathophysiology, combined with recognition of characteristic imaging patterns, is essential for accurate interpretation. This organ-based review summarized the key imaging features, differential diagnoses, and diagnostic pitfalls of diabetic complications. Increased awareness of these patterns will aid radiologists in making timely diagnoses, supporting multidisciplinary management, and ultimately improving patient outcomes in the growing diabetic population.

## Data Availability

No datasets were generated or analyzed during the current study.
